# Fear in the mind’s eye: the neural correlates of differential fear acquisition to imagined conditioned stimuli

**DOI:** 10.1093/scan/nsac063

**Published:** 2023-01-11

**Authors:** Lauryn Burleigh, Steven G Greening

**Affiliations:** Department of Psychology, Cognitive and Brain Sciences, Louisiana State University, Baton Rouge, LA 70803, USA; Department of Psychology, Cognitive and Brain Sciences, Louisiana State University, Baton Rouge, LA 70803, USA; Department of Psychology, Brain and Cognitive Sciences, University of Manitoba, Winnipeg, Manitoba R3T 2N2, Canada

**Keywords:** fear conditioning, mental imagery, fear, learning, emotion

## Abstract

Mental imagery is involved in both the expression and treatment of fear-related disorders such as anxiety and post-traumatic stress disorder. However, the neural correlates associated with the acquisition and generalization of differential fear conditioning to imagined conditioned stimuli are relatively unknown. In this study, healthy human participants (*n *= 27) acquired differential fear conditioning to imagined conditioned stimuli paired with a physical unconditioned stimulus (i.e. mild shock), as measured via self-reported fear, the skin conductance response and significant right anterior insula (aIn) activation. Multivoxel pattern analysis cross-classification also demonstrated that the pattern of activity in the right aIn during imagery acquisition was quantifiably similar to the pattern produced by standard visual acquisition. Additionally, mental imagery was associated with significant differential fear generalization. Fear conditioning acquired to imagined stimuli generalized to viewing those same stimuli as measured with self-reported fear and right aIn activity, and likewise fear conditioning to visual stimuli was associated with significant generalized differential self-reported fear and right aIn activity when imagining those stimuli. Together, the study provides a novel understanding of the neural mechanisms associated with the acquisition of differential fear conditioning to imagined stimuli and that of the relationship between imagery and emotion more generally.

## Introduction

There is a profound interaction between fear and mental imagery. Even at a young age, a child may become afraid of a non-existent monster, and adults may imagine that their thoughts or prayers are associated with acts of nature, such as thunder and lightning, or some otherwise uncontrollable life-or-death outcome (Muris *et al.*, 2003; Clasen, 2012; Singh, 2017). Anxiety and post-traumatic stress disorder (PTSD) are also associated with fear-related symptoms, elicited by mental imagery such as intrusive memories and flashbacks (Arntz *et al.*, 2007; Shin and Liberzon, 2010).

To better understand the relationship between mental imagery and fear, research has begun to use differential fear conditioning (Mertens *et al.*, 2020). In a standard differential fear-conditioning paradigm, a visually presented conditioned stimulus (CS+) is paired with an aversive unconditioned stimulus (US), and a second conditioned stimulus (CS−) is never paired with the US. As a result of the acquisition, the CS+ elicits a conditioned response (CR) of a larger magnitude than the CS−. The CR measured in human fear conditioning includes self-reported fear (LeDoux and Hofmann, 2018), skin conductance response (SCR) and brain activity measured using functional magnetic resonance imaging (fMRI). In a recent meta-analysis of differential fear conditioning in fMRI, Fullana *et al.* (2016) observed a significant fear-conditioning network that included the largest effects in the right and left anterior insula (aIn), respectively, as well as the dorsal anterior cingulate cortex (dACC) and dorsal medial prefrontal cortex (dmPFC). It is noteworthy that the amygdala did not display reliable differential responding according to this meta-analysis; however, the results summarized by Fullana *et al.* (2016) focused on univariate effects. Other research has demonstrated that multivoxel pattern analysis (MVPA) can be used to distinguish between CS+ and CS− trials based on the pattern of amygdala activity (Bach, *et al.*, 2011).

Several early studies provided evidence that mental imagery could be combined with fear conditioning as measured with SCRs (e.g. Yaremko and Werner, 1974; Holzman and Levis, 1991), and these studies often included limitations such as using outdated methods for quantifying the SCR, failing to demonstrate acquired differential conditioning and using between-group designs with too few participants. For example, Yaremko and Werner (1974) observed that participants who imagined both the CS+ (an auditory tone) and the US (a mild shock) together, compared to a group who imagined the US alone, had a higher frequency of SCRs in response to the offset of the CS+ during a generalization phase in which the auditory CS+ was physical presented. However, the study included no CS−, no description of whether SCRs were present during the trials involving imagery acquisition, no report of SCRs to the onset of the CS+, and no report of the magnitude of the SCRs. In perhaps the first study of differential conditioning in which an imagined CS+ was paired with a physical US (i.e. imagery acquisition), Holzman and Levis (1991) found that imagery of the CS+ produced significantly greater CRs (i.e. SCRs) during the early trials of an extinction test phase in the group that received the physical mild shocks paired with imagery of the CS+ compared to an unpaired group. However, the study did not report on the CRs to the CS− and, importantly, did not report any results from the acquisition phase.

More recently, Burleigh *et al.* (2022) addressed many of the limitations of these earlier studies and across two psychophysiological experiments and found robust evidence of successful differential fear learning produced by imagery acquisition training. Specifically, an imagined CS+ paired with a physical US (i.e. a mild shock) elicited a significantly greater self-reported fear of shock and SCR compared to an imagined CS− that was never paired with the US. Moreover, the magnitude of differential conditioning resulting from imagery acquisition for one pair of CSs was not significantly different from the magnitude of differential conditioning resulting from visual acquisition for a second pair of CSs (Burleigh *et al.*, 2022). Moreover, Burleigh *et al*. (2022) observed that following imagery acquisition, presentation of the visual CSs (which were never paired with the US) produced a significant differential fear generalization response. These generalization findings were consistent with a previous study that found that evaluative conditioning to imagined CSs generalizes to viewing the same stimuli as measured by reaction time (Lewis *et al.*, 2013). Yet, as neither Burleigh *et al*. (2022) nor any other previous studies have included fMRI, it is currently unknown whether differential imagery acquisition involving imagined CSs with a physical US is associated with significant activation of parts of the fear-conditioning network.

Other recent research has demonstrated that following successful acquisition of differential fear conditioning to visual or auditory CSs, mental imagery can be used in the expression (Burleigh *et al*., 2022; Greening *et al.*, 2022), regulation (Greening *et al.*, 2022), extinction (Agren *et al.*, 2017; Reddan *et al.*, 2018; Jiang and Greening, 2021), counter-conditioning (Koizumi *et al.*, 2016) and reconsolidation (Grégoire and Greening, 2019) of the acquired fear association. During acquisition itself, we recently observed that differential fear conditioning acquired to visual CSs generalizes to instances of imagining the respective CSs, reflected in greater self-reported fear, SCR and activation of the right aIn when imagining the CS+ than imagining the CS− (Greening *et al.*, 2022). However, Greening *et al.* (2022) did not evaluate whether imagined CSs themselves can acquire differential fear conditioning via pairing with a physical US as the US was physically presented when viewing the CS+ (i.e. visual acquisition), not when imagining the CS+ (i.e. imagery acquisition).

The primary aim of the current study was to determine if imagined CSs can be paired with a physical US, leading to a differential fear-conditioned response in brain regions classically associated with fear conditioning. We predicted that imagery acquisition would be associated with significantly more activity in areas of the fear-conditioning network when imagining the CS+ than imagining the CS− (Fullana *et al.*, 2016), specifically in the right aIn (Greening *et al.*, 2022), which would also be involved in visual acquisition. The secondary aim of the study was to determine the neural correlates associated with both the generalized differential response to viewing the CS+ *vs* the CS− following imagery acquisition (imagery-to-view generalization) and the generalized differential response to imagining the CS+ *vs* the CS− following visual acquisition (view-to-imagery generalization). Specifically, we began with a region-of-interest (ROI) analysis derived from Greening *et al.* (2022) to test both our primary and secondary predictions that significant differential generalization would be observed in the right aIn. These included both a univariate approach and an MVPA cross-classification approach to quantify the degree of pattern similarity between imagery acquisition and visual acquisition.

## Methods

### Participants

The study enrolled 31 healthy adults (17 females) between the ages of 18 and 33 (M = 24.31 years), all of which are included in the Neuroimaging section. The data of four participants were removed; two were non-responders (no detectable response after receiving a shock), and two included excessive noise in SCR data. Thus, a sample of 27 participants is included in the Self-Report Ratings of Fear and SCR sections. We also present the full set of self-report participant data, which includes those who were removed (Supplementary 2.2.3). The sample size was determined based on previous psychophysiological and behavioral research using the same experimental paradigm (Burleigh *et al.*, 2022); based on similar previous research in fMRI involving mental imagery generally (Dijkstra *et al.*, 2017) and based on research combining imagery and fear conditioning (Reddan *et al.*, 2018; Greening *et al.*, 2022). Participants were recruited through Louisiana State University’s (LSU’s) human participant study pool to participate in a 2 h MRI research session and provided written informed consent. The experiments were approved by LSU’s institutional review board. This study consisted of two phases: visual acquisition phase and imagery acquisition phase, which were counterbalanced across participants regarding which phase was presented first.

### Materials

Gabor patches (sinusoidal gratings) were used as the conditioned stimuli throughout the study. In the visual acquisition phase, a rightward (45° angle with lines from lower left to upper right corner) and a horizontal (0° angle with lines from left to right) Gabor patch were used as the CS+ and CS−, counterbalanced across participants. For the imagery acquisition phase, leftward (135° angle, lines from lower right to upper left corner) and vertical (90° angle with lines from bottom to top) Gabor patches were used for the CSs, counterbalanced across participants. Thus, each of the two phases involved a distinct pair of conditioned stimuli. The CSs were presented within the participants’ eight degrees of visual angle. On each trial, participants were provided an auditory instructional cue to either attend or imagine one of the Gabor patches. These trial-by-trial auditory instructions—‘imagine right’, ‘imagine horizontal’, ‘attend right’ and ‘attend horizontal’ in the visual acquisition phase and ‘imagine left’, ‘imagine vertical’, ‘attend left’ and ‘attend vertical’ in the imagery acquisition phase—were delivered using Sensimetric MRI-compatible insert earphones and were produced using www.fromtexttospeech.com (Greening *et al.*, 2022). Prior to each phase, participants were shown images of each Gabor patch included in the phase itself (Supplementary 1.1 and 1.2) but were not instructed which would be paired with the US, only that ‘a shock may occur’ during subsequent runs.

The US during the fear-conditioning trials was a mild electrical stimulation (administered using the STMISOC and STM100C modules of BIOPAC, Goleta, CA, USA). Two electrodes were placed on the fingertips of the first and second fingers of the non-dominant hand. The intensity of shock was individually set by each participant at a level that was ‘uncomfortable but not painful’ (M = 4.43 mA), as consistent with previous research (Greening *et al.*, 2016).

### Procedure and design

Once in the MRI scanner, participants completed two distinct phases: the visual acquisition phase and the imagery acquisition phase (see Supplementary 1.1 and 1.2 for the instructions). The phases were counterbalanced across participants regarding which was presented first. Each phase began with six habituation/practice runs followed by six fear acquisition runs. The purpose of the habituation/practice runs was first to habituate participants initial responses to the auditory cues and the Gabor patches (Jiang *et al.*, 2021). Second, the purpose was to familiarize participants with the task including providing participants with a suitable number of imagery trials on which to practice following our imagery cueing procedure. While the number of trials (see later) during the habituation/practice runs may seem relatively large compared to typical habituation phases, our design was consistent with previous research requiring participants to practice viewing and imagining the conditioned stimuli prior to a fear acquisition phase (Greening *et al.*, 2022), in which case the inclusion of practice trials did not prevent the acquisition of fear conditioning. Additionally, the data collected from the practice trials allowed for the independent evaluation of whether visual mental imagery generated significant visual cortex activity prior to the fear-conditioning acquisition phases (Kosslyn and Thompson, 2003; Dijkstra *et al.*, 2017). The participants also completed the Likert-style questionnaire after each phase (Supplementary 1.3 and 1.4).

**Fig. 1.**
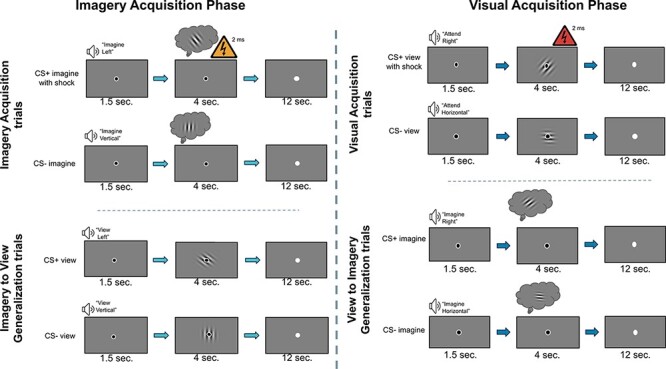
Trial structure. Participants imagined and viewed the patches associated with each of the visual acquisition and imagery acquisition phases, respectively. Top left: a CS+ view trial during visual acquisition with 2 ms mild electrical stimulation that co-terminates with the Gabor patch. Bottom left: a CS− view trial during visual acquisition. Top right: a CS+ imagine trial during imagery acquisition with 2 ms mild electrical stimulation that co-terminates with the 4 s imagery epoch. Bottom right: a CS− imagine trial during imagery acquisition.

The overall trial structure was identical for both the visual acquisition and imagery acquisition phases (Figure 1) and was adapted from recent psychophysiological research from the lab (Burleigh *et al.*, 2022). Each phase took ∼40 min to complete, including the habituation/practice runs, acquisition runs and the Likert-style questions.

#### Imagery acquisition phase

The imagery acquisition phase began with six habituation/practice runs, each of which consisted of eight trials. Each run contained two CS+ imagine without shock trials, two CS− imagine trials, two CS+ view trials and two CS− view trials. The trial order was fully randomized. Thus, participants viewed each CS 12 times and imagined each CS 12 times, for a total of 48 trials.

Next, participants completed six fear acquisition runs, each of which included 12 trials. Each run contained two CS+ imagine with shock trials (12 trials across all six runs), two CS+ imagine without shock trials (12 trials across all six runs), four CS− imagine trials (24 trials across all six runs), two CS+ view trials (12 trials across all six runs) and two CS− view trials (12 trials across all six runs). Thus, imagining CS+ was paired with the US 50% of the time and viewing the CSs was never paired with shock in this phase. In total, across the six runs, participants completed a total of 72 trials. The trial order was pseudo-random (Greening *et al.*, 2022). Each run began and ended with a CS− imagine trial. The first run in this phase also included a CS+ imagine with shock trial as the second trial to begin the fear association early. The second CS+ imagine with shock trial was randomly presented within the second half of the run. For the remaining three runs, the first CS+ imagine trial was randomly presented in the first half of the run and the second CS+ imagine trial was randomly presented in the second half of the run. Trials including a shock are not used in analyses to avoid any potential US confound in our modeling of CS+ trials. We also excluded the first and last CS− imagine trials of each run. This is the same approach used in Greening *et al.* (2022) and allowed us to avoid the potentially confounding orienting effect generated on the first trial of a run and allowed us to ensure that the number of trials in each first-level regressor was equal across conditions of interest for the analysis. All other trials not otherwise noted were randomized within the run.

#### Visual acquisition phase

The visual acquisition phase was similar to the imagery acquisition phase in structure, as it included six habituation/practice runs followed by six fear acquisition runs, but with several notable exceptions. First, a novel pair of conditioned stimuli was used. Second, each fear acquisition run included two CS+ view with shock trials, two CS+ view without shock trials, four CS− view trials, two CS+ imagine trials and two CS− imagine trials. In other words, only when viewing the CS+ did participants receive a shock (50% reinforcement rate), and no shock was ever delivered during imagery of the CSs.

### Self-reported measures

The self-reported measures were collected in a similar manner to previous research (Burleigh *et al*., 2022; Greening *et al.*, 2022). Briefly, at the end of each acquisition phase, participants completed a series of seven-point Likert-style questions to evaluate their (i) fear of getting shocked on each respective trial type (1, not at all, to 7, very much so); (ii) vividness of mental imagery on each respective imagery trial type (1, non-existent, to 7, very strong) and (iii) mental imagery effort on each respective imagery trial type (1, not at all, to 7, very hard). On each of these questions, no reference was ever made to the CS+ or CS− and, instead, participants were asked about the specific Gabor patches used in each phase (Supplementary 1.3 and 1.4).

### SCR collection and analysis

SCR was recorded during both acquisition phases but was not collected during either habituation/practice phases. The SCR was recorded at 2000 Hz sampling rates using the MP-150 system (BIOPAC). Two Ag/AgCl electrodes with a conductive saline-based gel (BIOPAC GEL101) were placed on the fingertips of the fourth and fifth fingers of the non-dominant hand. The SCR analyses were conducted in Matlab R2017a. The SCR was calculated by subtracting the baseline (average signal between 0 and 1 s) from the maximum peak amplitude during the 1–6 s time window following the CS onset. Each SCR was also required to be >0.02 microsiemens (µS). If these criteria were not met, the SCR was scored as zero. The trials including shocks were excluded in subsequent analyses (Jiang *et al.*, 2021; Jiang and Greening, 2021). SCRs were square-root transformed to attain normality prior to statistical analyses.

### Image acquisition

Subjects were scanned using a 3 T GE DISCOVERY MR750w MRI scanner with a 32-channel head coil. Each participant first received a high-resolution, T1-weighted, anatomical scan covering the whole brain (repetition time = 8.78 ms; echo time = 3.79 ms; field of view = 22.4 cm; voxel size = 3.5 mm isovoxels; 64 × 64 matrix). The following procedures were then followed for each phase.

Twenty-four fMRI scan runs (six habituation/practice and six acquisition runs for each phase) were run to measure blood-oxygenation-level-dependent (BOLD) changes. The fMRI images were taken with a T2*-gradient echo-planar imaging sequence (repetition time = 2000 ms; echo time = 25 ms; field of view = 22.4 cm; 64 × 64 matrix). Complete brain coverage was obtained with 76 slices of 3.5×3.5 mm in plane with a slice thickness of 3.5 mm, forming 3.5×3.5 × 3.5 mm voxels. Each functional run began with collecting three dummy volumes to account for T1 equilibrium effects, discarded as part of the pre-processing steps of data analysis. The total number of volumes per run, including dummy volumes, varied according to task type, but not phase: habituation/practice = 73 volumes (∼2.4 min/run) and acquisition = 109 volumes (∼3.6 min/run).

#### Functional MRI pre-processing

Both individual and group analyses were processed using FEAT (fMRI Expert Analysis Tool) in FMRIB’s Software Library (FSL, www.fmrib.ox.ac.uk/fsl) version 5.0.9. Registration of functional images to the high-resolution (T1-weighted) structural image and the standard space image was performed using FSL’s linear image regisration tool (FLIRT) (Jenkinson and Smith, 2001; Jenkinson *et al.*, 2002). The pre-statistics processing applied were as follows: motion correction FLIRT (Jenkinson *et al.*, 2002); slice-timing correction using Fourier-space time-series phase-shifting; non-brain removal using Brain Extraction Tool (BET) (Smith, 2002) spatial smoothing using a Gaussian kernel of full width half-maxium 7 mm; grand-mean intensity normalization of the entire 4D data set by a single multiplicative factor; and high-pass temporal filtering (Gaussian-weighted least-squares straight line fitting, with sigma = 50.0 s). The time-series statistical analysis was carried out using FSL’s Improved Linear Model function with local autocorrelation correction (Woolrich *et al.*, 2001).

#### Functional MRI univariate analysis

The data for both the ROI and whole-brain analyses were analyzed within the general linear model using a multi-level mixed-effects design. Each run was modeled separately at the single-participant level. Each condition of interest (i.e. onset of either viewing or imagining a given Gabor) was convolved using a double-gamma hemodynamic response function. We also modeled the CS+ reinforced (i.e. shock) trials and the first and last CS− trials as separate regressors, not used in higher-level analyses. A second-level analysis combined contrast estimates from the first level separately for each task (e.g. acquisition) for each participant using a fixed-effects model, forcing the random-effects variance to zero in FLAME (FMRIB’s Local Analysis of Mixed Effects; Beckmann *et al.* 2003; Woolrich *et al.*, 2004; Woolrich, 2008).

#### Region-of-interest analysis

To test a priori hypotheses regarding fear-conditioned generalization, we used a ROI small-volume correction analysis focused on the right aIn cluster observed in the neural overlap analysis of experiment 1 in Greening *et al.* (2022), which comprised an entirely independent participant sample. This approach ensured that the data used in the ROI selection were independent of the data being analyzed. The right aIn is also reliably activated in studies of human differential fear conditioning (Fullana *et al.*, 2016). The group-level analyses were carried out using FLAME stage 1 and stage 2 with automatic outlier detection (Beckmann *et al*., 2003; Woolrich *et al*., 2004; Woolrich, 2008) with pre-threshold masking by the right aIn map. The resulting *z* (Gaussianized T/F) statistic images were corrected for multiple comparisons using FSL’s cluster thresholding algorithm that applies Gaussian random field theory to estimate the probability of observing clusters of a given size. We applied a threshold of *z* > 2.3 and a (corrected) cluster size probability of *P* < 0.05 (Worsley, 2001).

#### Whole-brain analyses

The whole-brain group-level analyses were carried out using a similar approach to the ROI analysis but without pre-threshold masking. Specifically, we used FLAME (FMRIB’s Local Analysis of Mixed Effects) stage 1 and stage 2 with automatic outlier detection (Beckmann *et al*., 2003; Woolrich, 2008; Woolrich *et al*., 2004). The resulting *z* (Gaussianized T/F) statistic images were corrected for multiple comparisons using FSL’s cluster thresholding algorithm that applies Gaussian random field theory to estimate the probability of observing clusters of a given size. We applied a threshold of *z* > 2.3 and a (corrected) cluster size probability of *P* < 0.05 (Worsley, 2001).

#### MVPA cross-classification

To quantify the degree of informational similarity between key conditions, in our study we adapted an MVPA cross-classification approach used previously (Kaplan *et al.*, 2015; Greening, Mitchell, Smith, 2018; Greening *et al.*, 2022). We used an ROI approach involving the right aIn consistent with the univariate ROI analysis, as well as an exploratory bilateral amygdala ROI analysis. Our primary purpose for this analysis was to evaluate whether a classifier trained on the pattern of activity in our ROIs elicited during imagine CS+ *vs* imagine CS− trials from the imagery acquisition phase could predict whether participants were viewing the CS+ *vs* CS− during the visual acquisition phase, and vice versa. Our secondary purpose was to evaluate the pattern similarities between imagery and view trials within each of the respective acquisition phases. For example, to quantify pattern similarity between imagery acquisition and imagery-to-view generalization, we trained a classifier on trials from the former and tested on trials from the latter. We did likewise to compare visual acquisition trials to the visual-to-imagery generalization trials.

Pre-processing and first-level modeling of the fMRI data used for the MVPA cross-classification analysis were identical to the univariate analysis except that each trial was modeled independently in the first-level general linear model. As with the univariate analysis, the MVPA used only CS+ trials without shock. Additionally, to ensure an equal number of CS+ and CS− trials during both the training and testing of our classifiers, we excluded the first and last CS− trial from each run. Thus, we were able to extract trial-wise normalized parameter estimates from the voxels in our ROIs. Regarding the ROIs, the right aIn masks were the same as the ones used in the aforementioned univariate ROI analyses, which were taken from the study of Greening *et al.* (2022). For the right aIn we extracted all voxels from the ROI and included them as the features in the primary cross-classification analyses. For the exploratory bilateral amygdala analyses we used the same methods as used in previous research (Greening *et al.*, 2022). Specifically, a bilateral amygdala mask was created by combining the right and left anatomical amygdala masks from the Harvard-Oxford atlas, thresholded at 50%. Additionally, consistent with Bach *et al.*, (2011) only the 300 voxels with the largest univariate signal were selected as the features for a given participant’s classifier training. Finally, the MVPA used support vector machine (SVM) classification implemented with the PyMVPA toolbox (Hanke *et al.*, 2009) at the single-participant level, after which the within-participant results were aggregated for group-level analysis.

To determine whether our group-level classification accuracies were significant, we compared each of them to respective group-level null distributions produced for each ROI by combining permutation testing and bootstrapping (Stelzer *et al.*, 2013; Greening *et al.*, 2018, 2008). Specifically, we ran 10 000 iterations per participant in which we trained an SVM on data with randomly permuted target labels from the training set and tested on the held-out testing data. Afterwards, a group-level null distributions were produced using a random sampling with replacement bootstrapping procedure with 10 000 iterations, such that a group mean accuracy estimate was generated at each iteration (i.e. we randomly selected one element from each participant’s previously computed individual null distribution with replacement during each iteration). This procedure allowed us to also confirm that the chance performance for all estimated null distributions was ∼50%.

#### Independent evaluation of visual imagery

To further confirm the use of visual mental imagery by the participants, we made use of the habituation/practice data. To do so, we first contrasted both imagery conditions *vs* baseline for each of the two phases and conducted the group-level analyses using the same methods described previously. This resulted in two independent whole-brain univariate maps. The first map was derived from the habituation/practice trials of the visual acquisition phase, in which participants imagined the rightward and horizontal Gabor patches. The second map was from the habituation/practice trials of the imagery acquisition phase, in which participants imagined the leftward and vertical Gabor patches.

Next, we performed a statistical conjunction analysis (Nichols *et al.*, 2005) using the easythresh_conj function. The inputs to the function were the two uncorrected brain maps produced earlier. This resulted in a group-level whole-brain conjunction map, which was thresholded and cluster-corrected similar to the whole-brain analysis described previously. Specifically, the group-level map was voxel-thresholded to *z* > 2.3 and cluster-corrected to *P* < 0.05.

This analysis allowed us to determine whether visual cortex activity was elicited when visual imagery was performed during the habituation/practice trials, similar to previous studies of visual imagery (Dijkstra *et al.*, 2017). We were then able to use the results as a functional localizer for a visual cortex ROI that could be interrogated during the subsequent fear acquisition trials, which was not confound by associated learning.

Importantly, the conjunction analysis revealed a significant cluster of activity that included an aspect of the ventral visual cortex, but that was part of a larger cluster that extended all the way into the dorsal and lateral parietal lobe. We isolated the visual cortex voxels by first eroding the larger cluster till the visual cortex cluster was disconnected from the rest of the cluster, and then we dilated the resulting visual cortex map by the same erosion factor. Next, this visual cortex cluster was used as an independent ROI for our analysis of the fear acquisition trials from each of the imagery acquisition and visual acquisition phases. This allowed us to evaluate whether mental imagery during each of the two acquisition phases was associated with a significant amount of univariate visual cortex activity above baseline.

## Results

### Self-report ratings of fear

**Fig. 2.**
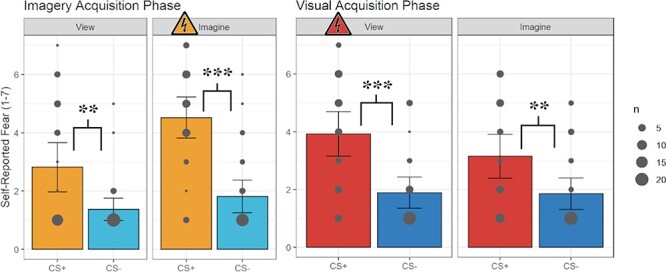
Self-reported fear results. Self-reported fear was collected using a seven-point Likert-style questionnaire (1, not at all; 7, very much so) for the viewing and imagining conditions during the imagery acquisition phase (left) and the visual acquisition phase (right). The size of each dot indicates how many participants chose each value. Error bars represent 95% confidence intervals. The shock symbol indicates which condition participants were fear-conditioned to in each acquisition phase. Asterisks indicate significant *P*-values resulting from the *t*-test results for each key comparison relating to the study’s primary predictions. ANOVA results and additional analyses can be found in the written results. * = *P* < 0.05, ** = *P* < 0.01, and *** = *P* < 0.001.

A 2 × 2 × 2 repeated measures analysis of variance (ANOVA) involving CS type (CS+ or CS−) by percept modality (view or imagine) by phase (visual acquisition or imagery acquisition) was conducted (Figure 2; Supplementary 2.2.2). There was a significant three-way interaction, *F*(1, 26) = 7.24, *P* = 0.012, η^2^ = 0.023, and an interaction between percept modality and phase, *F*(1, 26) = 17.56, *P* < 0.001, η^2^ = 0.050. Finally, there was a main effect of CS type, *F*(1, 26) = 39.90, *P* < 0.001, η^2^ = 0.010. No other statistical significance was found. Importantly, the results of the supplementary analysis on the full sample of self-reported fear data are inferentially identical (Supplementary 2.2.3). In the following we unpack the critical three-way interaction in a manner related to the primary hypothesis and two secondary hypotheses.

Beginning with fear acquisition, in the imagery acquisition phase participants had greater self-reported fear when imagining the CS+ than when imagining the CS−, *t*(26) = 6.11, *P* < 0.001, *d* = 1.18. Similarly, in the visual acquisition phase, participants reported more fear both when viewing the CS+ and viewing the CS−, *t*(26) = 4.79, *P* < 0.001, *d* = 0.92.

Regarding our secondary hypotheses concerning generalization, in the imagery acquisition phase when viewing the CS+ *vs* viewing the CS−, *t*(26) = 3.54, *P* = 0.002, *d* = 0.68, as well as in the visual acquisition phase, participants reported more fear when imagining the CS+ than imagining the CS−, *t*(26) = 3.51, *P* = 0.002, *d* = 0.86.

To further understand the three-way interaction, we used follow-up *t*-tests between the various conditions. Within the visual acquisition phase, the viewed CS+ was not significantly greater than the imagined CS+, *t*(26) = 2.03, *P* = 0.052, *d* = 0.39. Conversely, in the imagery acquisition phase, the imagined CS+ was greater than the viewed CS+, *t*(26) = 4.13, *P* < 0.001, *d* = 0.79, and the imagined CS− was also greater than the viewed CS−, *t*(26) = 2.37, *P* = 0.025, *d* = 0.46. Comparing between the visual and imagery acquisition phases, there was greater self-reported fear when conditioning to the viewed CS+ (visual acquisition phase) than generalizing to the viewed CS+ (imagery acquisition phase), *t*(26) = 2.11, *P* = 0.045, *d* = 0.41. This was similarly the case for the viewed CS− in both phases (i.e. viewed CS− from the visual acquisition phase *vs* viewed CS− from the imagery acquisition phase), *t*(26) = 2.48, *P* = 0.020, *d* = 0.48. There was also significantly greater fear reported when participants were conditioned to the imagined CS+ (imagery acquisition phase) than generalizing to the imagined CS+ (visual acquisition phase), *t*(26) = 3.16, *P* = 0.004, *d* = 0.61. Finally, the generalized fear to the imagined CS− (visual acquisition phase) was greater than the generalized fear to the viewed CS− (imagery acquisition phase), *t*(26) = 2.30, *P* = 0.030, *d* = 0.06. All other comparisons were non-significant (Supplementary 2.2.5).

To rule out any potential order effects with respect to the two different phases, we also ran follow-up analyses with order as an additional factor. We found no significant main effect of order, nor any significant interactions involving order (see Supplementary 2.2.3 and 2.2.4 for details).

#### Bayesian analysis self-reported fear data

Regarding acquiring fear to imagined stimuli (i.e. imagery acquisition phase, imagining the CS+ and imagining the CS−), the Bayesian (Bayes) analysis resulted in very strong, decisive evidence for H_1_ relative to H_0_, BF_10_(0.707) > 1000.00. Regarding acquiring fear to visual stimuli (i.e. visual acquisition phase, viewing the CS+ and viewing the CS−), the Bayes analysis resulted in very strong, decisive evidence for H_1_ relative to H_0_, BF_10_(0.707) > 429.75.

When assessing fear generalizing from an imagined to a viewed stimulus (i.e. imagery acquisition phase, viewing the CS+ and viewing the CS−), the Bayes analysis resulted in very strong evidence for H_1_ relative to H_0_, BF_10_(0.707) = 23.59. Regarding fear generalizing from a viewed to an imagined stimulus (i.e. visual acquisition phase, imagining the CS+ and imagining the CS−), the Bayes analysis resulted in very strong evidence for H_1_ relative to H_0_, BF_10_(0.707) = 22.02.

See Supplementary 2.2.6 for corresponding sensitivity analyses.

#### Self-report ratings of imagery effort and vividness

Self-reported imagery effort and vividness were evaluated using two independent 2 × 2 ANOVAs using CS type (CS+ or CS−) and phase (visual acquisition or imagery acquisition) for each. The imagery effort ANOVA found a significant interaction, *F*(1, 26) = 8.16, *P* = 0.008, η^2^ = 0.015 (Supplementary 2.4). To unpack the interaction, follow-up tests were performed; however, all possible combinations of *t*-tests were found to be not significant, and there were trends towards significance for imagery acquisition imagine CS+ (range = 1–7, median = 6.00) greater than imagine CS− (range = 1–7, median = 6.00), t(26) = 2, *P* = 0.056, *d* = 0.38, and imagery acquisition imagine CS− greater than visual acquisition imagine CS−, *t*(26) = 1.86, *P* = 0.075, *d* = 0.36 (visual acquisition CS+: range = 1–7, median = 5.00; and visual acquisition CS−: range = 1–7, median = 5.00).

Conversely, the imagery vividness ANOVA reported no significant findings (Supplementary 2.3; imagery acquisition CS+: range = 1–7, median = 6.00; imagery acquisition CS−: range = 2–7, median = 6.00; visual acquisition CS+: range = 1–7, median = 5.00; and visual acquisition CS−: range = 2–7, median = 5.00).

### SCR data

**Fig. 3.**
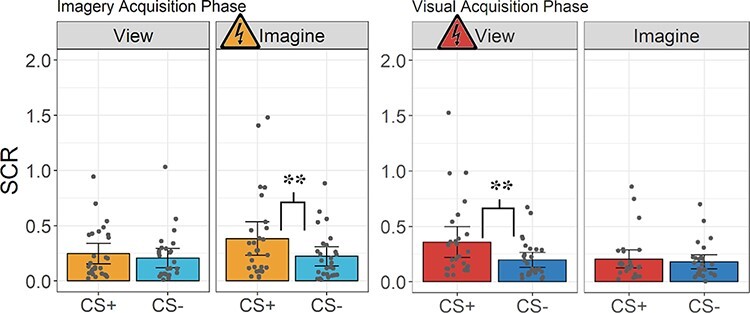
SCR results. SCR is reported on the *y*-axis. Each dot on the graphs represents a participant’s mean SCR for a given condition during the imagery acquisition phase (left) and the visual acquisition phase (right). Error bars represent 95% confidence interval. The shock symbol indicates which condition participants were fear-conditioned to in each acquisition phase. Asterisks indicate significant *P*-values resulting from the *t*-test results for each key comparison relating to the study’s primary predictions. ANOVA results and additional analyses can be found in the written results. * = *P* < 0.05, ** = *P* < 0.01, and *** = *P* < 0.001.

A 2 × 2 × 2 repeated measures ANOVA involving CS type (CS+ or CS−) by percept modality (view or imagine) by phase (visual acquisition or imagery acquisition) was also conducted on the SCR data (see Supplementary 2.5.1 for descriptive statistics). There was a significant three-way interaction, *F*(1, 26) = 8.37, *P* = 0.008, η^2^ = 0.016, and an interaction between percept modality and phase, *F*(1, 26) = 8.93, *P* = 0.006, η^2^ = 0.025. Finally, there was a main effect of CS type, *F*(1, 26) = 14.68, *P* < 0.001, η^2^ = 0.036. No other statistical significance was found (Figure 3; Supplementary 2.5.2 for ANOVA table). To unpack the three-way interaction, we proceeded starting with comparisons most relevant to our study hypotheses.

Critically, regarding fear acquisition in the imagery acquisition phase, participants had a larger SCR when imagining the CS+ than when imagining the CS−, *t*(26) = 3.26, *P* = 0.003, *d* = 0.63. In the visual acquisition phase, during acquisition, participants also showed a greater SCR when acquiring fear of viewing the CS+ than viewing the CS−, *t*(26) = 3.07, *P* = 0.005, *d* = 0.59.

Regarding generalization, in the imagery acquisition phase, there was no significant difference in the generalized SCR when viewing the CS+ *vs* viewing the CS−, *t*(26) = 1.17, *P* = 0.252, *d* = 0.23. In the visual acquisition phase, there was also no difference in SCR when imagining the CS+ *vs* imagining the CS−, *t*(26) = 1.23, *P* = 0.230, *d* = 0.24.

Within the imagery acquisition phase, the SCR was significantly greater for the imagined CS+ than the viewed CS+, *t*(26) = 2.38, *P* = 0.025, *d* = 0.46. Within the visual acquisition phase, the viewed CS+ SCR was greater than the imagined CS+ SCR, *t*(26) = 3.30, *P* = 0.003, *d* = 0.63. Between phases, the CR to the imagined CS+ in the imagery acquisition phase was greater than the generalization response to the imagined CS+ from the visual acquisition phase, *t*(26) = 2.56, *P* = 0.017, *d* = 0.49. All other comparisons were non-significant (Supplementary 2.5.5).

To rule out any potential order effects, we also ran follow-up analyses with order as an additional factor. We found no significant main effect of order, nor any significant interactions involving order (see Supplementary 2.5.3 and 2.5.4 for details).

#### Bayes analysis SCR data

Regarding acquiring fear to imagined stimuli (i.e. imagery acquisition phase, imagining the CS+ and imagining the CS−), the Bayes analysis resulted in strong evidence for H_1_ relative to H_0_, BF_10_(0.707) = 12.56. Regarding acquiring fear of visual stimuli (i.e. visual acquisition phase, viewing the CS+ and viewing the CS−), the Bayes analysis resulted in substantial evidence for H_1_ relative to H_0_, BF_10_(0.707) = 8.54.

When assessing fear generalizing from an imagined to a viewed stimulus (i.e. imagery acquisition phase, viewing the CS+ and viewing the CS−), the Bayes analysis resulted in anecdotal evidence for H_0_ relative to H_1_, BF_01_(0.707) = 2.65. Regarding fear generalizing from a viewed to an imagined stimulus (i.e. visual acquisition phase, imagining the CS+ and imagining the CS−), the Bayes analysis resulted in anecdotal evidence for H_0_ relative to H_1_, BF_01_(0.707) = 2.49.

See Supplementary 2.5.6 for sensitivity analyses.

### Neuroimaging

#### Fear acquisition

**Fig. 4.**
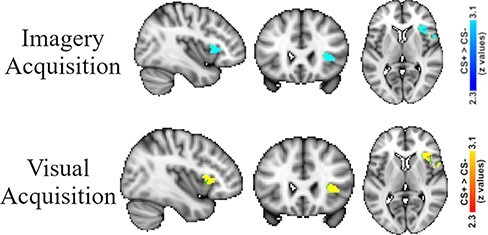
Functional MRI results during differential fear acquisition using small-volume correction in the right aIn ROI from Greening*et al*. (2022). Top row: results from imagining the CS+ > imagining the CS− during the imagery acquisition phase reveal significant differential acquisition in the right aIn. Bottom row: Results from viewing the CS+ > viewing the CS− during the visual acquisition phase reveal significant differential acquisition in the right aIn. The significant clusters are viewed within the right aIn ROI, and the ROI voxels that were not activate above threshold are included in the figure (i.e., the green voxels).

Fear acquisition was analyzed using the right aIn ROI mask from Greening *et al.* (2022). Both the imagery fear acquisition analysis (i.e. imagine CS+ *vs* imagine CS− from the imagery acquisition phase) and visual fear acquisition analysis (i.e. view CS+ *vs* view CS− from the visual acquisition phase) revealed significant activation within the ROI (Figure 4—top row: imagery fear acquisition and bottom row: visual fear acquisition; Table 1).

**Table 1.** Univariate ROI results

					MNI		
^a^	*k*	Brain region	H	*z*	X	Y	Z
*Imagery acquisition phase: imagine CS+ > imagine CS−*							
1	248	Anterior insula	R	3.82	40	24	6
*Imagery acquisition phase: imagine CS− > imagine CS+*							
		No significant clusters					
*Imagery-to-view generalization: view CS+ > view CS−*							
1	54	Anterior insula	R	3.02	38	26	2
*Imagery-to-view generalization: view CS− > view CS+*							
		No significant clusters					
*Visual acquisition phase: view CS+ > view CS−*							
1	185	Anterior insula	R	3.52	36	26	4
2	23	Anterior insula	R	2.87	54	6	6
*Visual acquisition phase: view CS− > view CS+*							
		No significant clusters					
*View-to-imagery generalization: imagine CS+ > imagine CS−*							
1	17	Anterior insula	R	2.45	34	26	8
*View-to-imagery generalization: imagine CS− > imagine CS+*							
		No significant clusters					

a = the cluster number, ordered by size; *k* = number of contiguous voxels in the cluster; Brain region = the region(s) in the cluster (region names are taken from the Harvard-Oxford atlas in FSL); H = principal hemisphere of the cluster, right (R) or left (L); *z* = maximum *z*-value from the cluster within the given brain region; MNI (X, Y, Z) = coordinates of the voxel with the maximum effect in the standardized space of the Montreal Neurological Institute (MNI), represented in units of millimeters (mm).

**Fig. 5.**
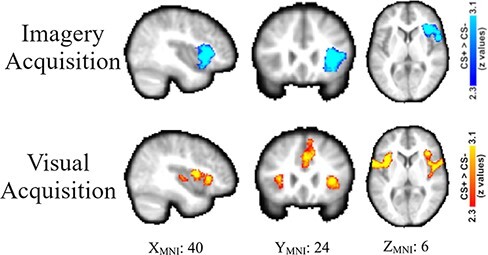
Functional MRI results during differential fear acquisition. Top row: whole-brain results from the imagery acquisition phase when fear conditioning to an imagined stimulus CS+ > CS−. Bottom row: whole-brain results from the visual acquisition phase when fear conditioning to a visually stimulus CS+ > CS−.

We also performed whole-brain fear acquisition analyses. The whole-brain analysis of imagine CS+ *vs* imagine CS− from the imagery acquisition phase revealed significantly greater activity in a right-hemisphere cluster that included the aIn, frontal operculum and inferior frontal gyrus (Figure 5—top row; Table 2). A whole-brain analysis also revealed significantly greater activity in the precentral gyrus when imagining the CS− than when imagining the CS+.

**Table 2.** Fear acquisition whole-brain results

					MNI		
^a^	*k*	Brain region	H	*z*	X	Y	Z
*Imagery acquisition phase: imagine CS+ > imagine CS−*							
1	2076	Anterior insula, frontal operculum, inferior frontal gyrus	R	4.30	36	14	−14
*Imagery acquisition phase: imagine CS− > imagine CS+*							
1	4312	Precentral gyrus	L	4.81	−52	−8	38
*Visual acquisition phase: view CS+ > view CS−*							
1	4233	Dorsal anterior cingulate cortex, dorsal medial prefrontal cortex	R/L	5.53	−2	6	38
2	1608	Posterior parietal cortex	R	5.08	58	−26	26
3	1378	Anterior insula	R	4.77	50	−2	12
4	1340	Anterior insula	L	4.16	−62	6	6
5	1329	Posterior parietal cortex	L	4.96	−58	−30	28
*Visual acquisition phase: view CS− > view CS+*							
		No significant clusters					

a = the cluster number, ordered by size; *k* = number of contiguous voxels in the cluster; Brain region = the region(s) in the cluster (region names are taken from the Harvard-Oxford atlas in FSL); H = principal hemisphere of the cluster, right (R) or left (L); *z* = maximum *z*-value from the cluster within the given brain region; MNI (X, Y, Z) = coordinates of the voxel with the maximum effect in the standardized space of the Montreal Neurological Institute (MNI), represented in units of millimeters (mm).

The whole-brain analysis of the view CS+ *vs* view CS− from the visual acquisition phase revealed robust activity in multiple regions of the fear-conditioning network. For example, differential activity (CS+ > CS−) was observed in the bilateral aIn and the dACC/dmPFC (Figure 5—bottom row; Table 2).

#### Differences in differential activity for visual acquisition vs imagery acquisition

This analysis revealed no significant clusters of activation.

#### Fear generalization

##### Imagery-to-view generalization (imagery acquisition phase)

**Fig. 6.**
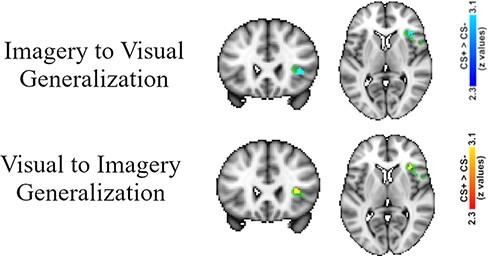
Greening*et al*. (2022). Top row: results from viewing the CS+ > viewing the CS− during the imagery acquisition phase reveal significant differential generalization in the right aIn. Bottom row: results from imagining the CS+ > imagining the CS− during the visual acquisition phase reveal significant differential generalization in the right aIn. The significant clusters are viewed within the right aIn ROI, and the ROI voxels that were not activate above threshold are included in the figure (i.e., the green voxels).

Our small-volume ROI analysis revealed significant activity in the right aIn when viewing the CS+ *vs* viewing the CS− during the imagery acquisition phase (Figure 6; Table 1).

##### View-to-imagery generalization (visual acquisition phase)

Our Greening *et al.* (2022) ROI analysis revealed significant activity in the right aIn, this time when imagining the CS+ *vs* imagining the CS− during the visual acquisition phase (Figure 6; Table 1).

#### MVPA cross-classification

##### Right aIn cross-classification

**Fig. 7.**
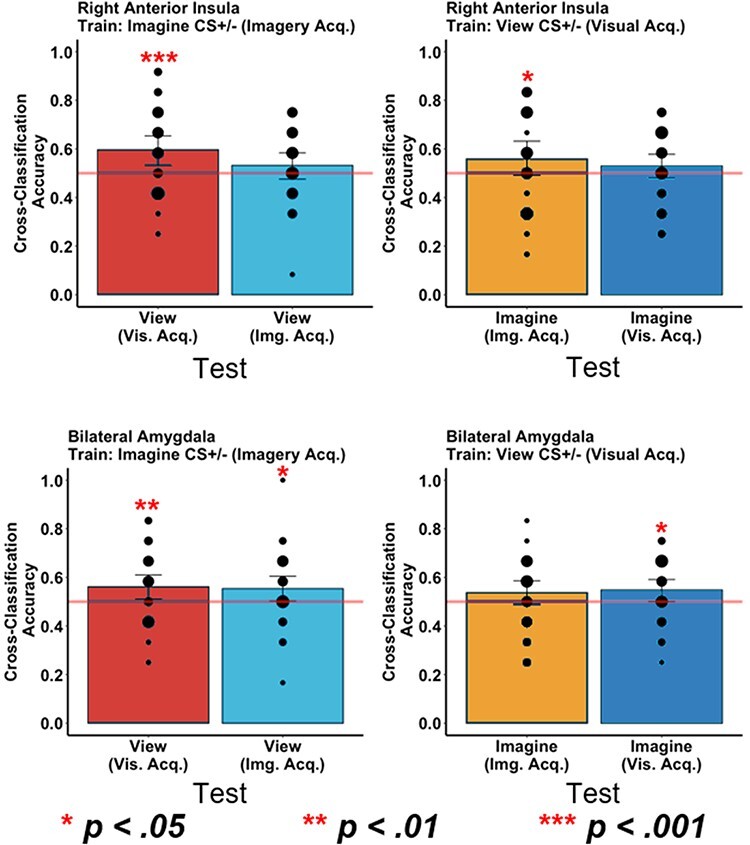
MVPA cross-classification results from the right aIn ROI (top row) and the bilateral anatomical amygdala mask (bottom row). The red horizontal line represents chance classification accuracy (50%), and the black dots represent the number of participants with a given classification accuracy (smaller dots *n* = 2; larger dots *n* = 4).

First, we extract the patterns of activity from the right aIn ROI from Greening *et al.* (2022) for our MVPA cross-classification analyses. Importantly, the classifier trained on the imagery trials of imagery acquisition phase (imagine CS+ non-reinforced trials *vs* imagine CS− trials) revealed significant cross-classification of view trials from visual acquisition trials (view CS+ *vs* view CS− trials) above chance, *P* < 0.001 (Figure 7). Likewise, the classifier trained on the view trials of the visual acquisition (view CS+ non-reinforced trials *vs* view CS− trials) also revealed significant cross-classification of imagine trials from the imagery acquisition phase (imagine CS+ *vs* imagine CS− trials) above chance, *P* = 0.018 (Figure 7).

However, regarding generalization, the classifier trained on the imagery trials of imagery acquisition was unable to classify the view trials of the imagery acquisition phase (i.e. the imagery-to-view generalization trials) above chance, *P *= 0.093 (Figure 7); however, there may be a trend. Moreover, the classifier trained on the view trials of visual acquisition was unable to cross-classify the imagery trials of the visual acquisition phase (i.e. the view-to-imagery generalization trials) above chance, *P* = 0.11 (Figure 7).

##### Bilateral amygdala

Next, we extract the pattern of activity from the anatomical bilateral amygdala ROI. Notably, on the one hand, the classifier trained on the imagery trials of the imagery acquisition phase (imagine CS+ non-reinforced trials *vs* imagine CS− trials) revealed significant cross-classification of view trials from visual acquisition trials (view CS+ *vs* view CS− trials) above chance, *P* = 0.004 (Figure 7). On the other hand, the classifier trained on the view trials of the visual acquisition (view CS+ non-reinforced trials *vs* view CS− trials) did not reveal significant cross-classification of imagine trials from the imagery acquisition phase (imagine CS+ *vs* imagine CS− trials) above chance, *P* = 0.066 (Figure 7); however, this value may reflect a trend towards significant.

Interestingly, we also observed significant cross-classification effects with respect to generalization. Specifically, the classifier trained on the imagery trials of imagery acquisition significantly cross-classified the view trials of the imagery acquisition phase (i.e. the imagery-to-view generalization trials) above chance, *P *= 0.017 (Figure 7). Similarly, the classifier trained on the view trials of visual acquisition was able to cross-classify the imagery trials of the visual acquisition phase (i.e. the view-to-imagery generalization trials) significantly above chance, *P* = 0.015 (Figure 7).

#### Independent evaluation of visual imagery

##### Conjunction analysis of imagery trials during habituation/practice

**Fig. 8.**
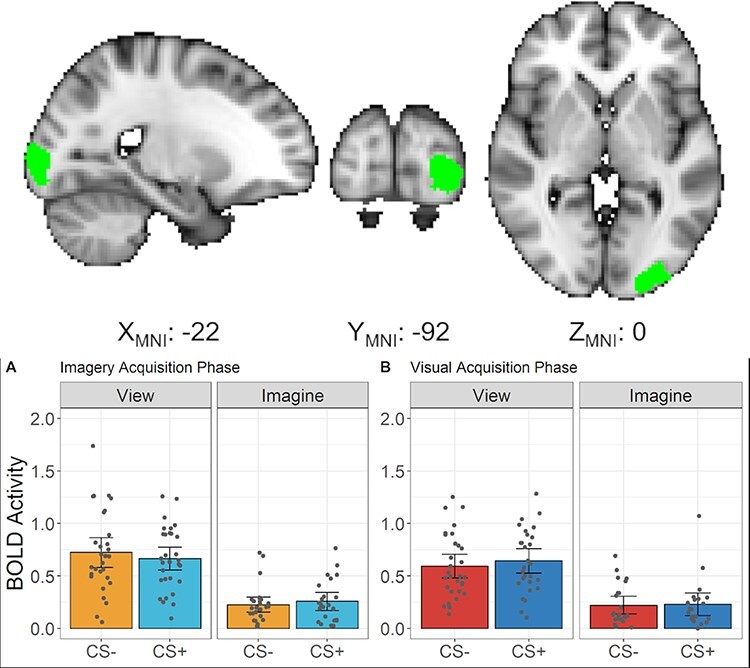
Top - Visual cortex ROI produced from the conjunction analysis of imagery trials from the habituation/practice runs of both the imagery acquisition phase and the visual acquisition phase. Bottom - plots of the univariate BOLD response (percent signal change) during the acquisition phase of both imagery acquisition (bottom-left) and the visual acquisition phases (bottom-right). Each dot indicates an individual participant’s BOLD response. Error bars represent 95% confidence intervals.

The whole-brain conjunction analysis of all imagery trials *vs* baseline from the habituation/practice runs from both the imagery acquisition phase and the visual acquisition phase revealed significant activation across a network of regions commonly observed in visual imagery (Dijkstra *et al.*, 2017; Moen *et al.*, 2020). Specifically, we observed bilateral activity aspects of the frontoparietal cortex including in the middle frontal gyrus, superior frontal gyrus, angular gyrus and superior parietal lobe (Table 3; Supplementary 2.6.1). Most notably, we observed significant activity in parts of the visual cortex including the occipital pole and the lateral occipital cortex (Figure 8; Table 3). This visual cortex region was then used as an ROI for evaluating whether imagery during the actual acquisition runs also produced significant visual cortex activity.

**Table 3.** Whole-brain conjunction of imagery *vs* baseline trials from the imagery habituation/practice runs

					MNI		
^a^	*k*	Brain region	H	*z*	X	Y	Z
*Imagery of rightward and horizontal Gabor patches (IA) ∩ imagery of leftward and vertical Gabor patches*							
1	12 685	Frontal pole, precentral gyrus, middle frontal gyrus, superior frontal gyrus, supplementary motor cortex, insular cortex, frontal operculum, temporal pole, striatum	R/L	5.13	54	10	8
2	5652	Lateral occipital cortex (inferior), occipital pole, lateral occipital cortex (superior), superior parietal lobe, supramarginal gyrus, angular gyrus	L	6.71	−42	−54	54
3	2941	Lateral occipital cortex (superior), superior parietal lobe, supramarginal gyrus, angular gyrus	R	5.43	40	−52	58

a = the cluster number, ordered by size; *k* = number of contiguous voxels in the cluster; Brain region = the region(s) in the cluster (region names are taken from the Harvard-Oxford atlas in FSL); H = principal hemisphere of the cluster, right (R) or left (L); *z* = maximum *z*-value from the cluster within the given brain region; MNI (X, Y, Z) = coordinates of the voxel with the maximum effect in the standardized space of the Montreal Neurological Institute (MNI), represented in units of millimeters (mm).

##### Visual cortex ROI analysis on imagery trials during acquisition

Using the visual cortex ROI, compared to baseline, there was significant activity in the visual cortex during the imagery trials for both the imagery acquisition runs, t(30) = 4.19, *P *< 0.001 (Figure 8), and the visual acquisition runs, t(30) = 2.47, *P *= 0.019 (Figure 8).

## Discussion

The primary aim of the current study was to determine if mental imagery of basic objects interacts with a physical US, thereby leading to differential fear conditioning in multiple measures including fMRI. Consistent with our primary hypothesis that differential fear conditioning can be established to imagined percepts, the imagery acquisition phase revealed significant fear conditioning to imagined percepts (imagery acquisition CS+ > CS−) in the self-reported fear, SCR and right aIn activity measured with fMRI.

The self-reported fear and SCR results for imagery acquisition were both confirmed using both null hypothesis statistics testing (NHST) and Bayes analyses, which both showed significant differential fear conditioning for imagined percepts, consistent with previous research (Holzman and Levis, 1991; Burleigh *et al.*, 20212). Importantly, our results extended the existing literature by revealing significant activation of the right aIn during mental imagery of the CS+ *vs* the CS− during imagery acquisition using an independent ROI mask from Greening *et al.* (2022). The involvement of the right aIn was further confirmed in the whole-brain analysis. It is tempting to speculate that there is something important about the lateralized right aIn effect observed in the whole-brain results of the imagery acquisition phase, given that previous research involving mental imagery and fear conditioning has found a similar lateralized response (Reddan *et al.*, 2018; Greening *et al.*, 2022). However, other recent research involving mental imagery and fear, though not fear conditioning *per se*, found significant bilateral aIn activity during fear-related *vs* neutral imagery with larger effect sizes in the left aIn (Hoppe *et al.*, 2021). Additionally, the whole-brain analysis from the visual acquisition runs revealed significant view CS+ > view CS− activity in multiple brain regions associated with differential fear conditioning including bilateral aIn and dACC (Fullana *et al.*, 2016).

The results from our MVPA cross-classification analyses further quantified the degree of pattern similarity observed in the right aIn during both imagery acquisition and visual acquisition. Specifically, a classifier trained on the pattern of right aIn activity during imagining CS+ (non-reinforced) *vs* CS− trials during imagery acquisition could be used to significantly decode whether participants were viewing the CS+ (non-reinforced) *vs* CS− during visual acquisition, and vice versa. A similar result was also observed using the bilateral amygdala ROI such that the classifier trained on imagery acquisition trials could significantly classify the visual acquisition trials; however, the opposite was not true. Specifically, the classifier trained on visual acquisition trials was unable to decode imagery acquisition trials significantly above chance. Nevertheless, these results are the first to demonstrate that there is significant pattern similarity between imagined conditioned and visual conditioned stimuli in both the aIn and amygdala. These results are also consistent with previous research that used whole-brain MVPA cross-classification to demonstrate the effect of mental imagery extinction (Reddan *et al.*, 2018). Moreover, like our results, Reddan *et al.* (2018) observed that voxels from the insula and amygdala made a reliable contribution to their classifier.

The secondary aim of the study was to evaluate the neural correlates associated with the generalization of differential fear conditioning from imagery acquisition to viewing the conditioned stimuli and from visual acquisition to imagery of the conditioned stimuli. Regarding generalization from imagining to viewing the percept (i.e. imagery-to-view generalization), self-reported fear indicated significant differential fear generalization to viewing the CS+ *vs* viewing the CS− according to both NHST and Bayes analyses. This comparison also produced significant right aIn activation using a small-volume ROI analysis. Moreover, our MVPA cross-classification analysis involving the bilateral amygdala ROI also revealed that a classifier trained on the imagery trials could significantly decode the view generalization trials, despite a lack of univariate effects involving the amygdala (Bach *et al.*, 2011). Conversely, both the SCR analysis and the MVPA cross-classification analysis involving the right aIn failed to show significant differential visual generalization after imagery acquisition CS+ > CS−. Nevertheless, the general findings of differential conditioning generalization from imagery acquisition to visual generalization are consistent with previous research (Holzman and Levis, 1991; Burleigh *et al.*, 20212). However, this is the first study to reveal significant brain activity during visual generalization following imagery acquisition.

Similar findings were observed during imagery generalization following the visual acquisition of differential conditioning. Specifically, self-reported fear indicated significant differential fear generalized to imagined CS+ *vs* CS− according to both NHST and Bayes analyses. Importantly, we also observed significant activity in the right aIn when imagining the CS+ *vs* imagining the CS− during the visual acquisition phase. Furthermore, we again observed significant cross-classification in the bilateral amygdala such that a classifier trained on the view trials could significantly decode the imagery generalization trials, though this cross-classification effect was not observed in the insula ROI. On the other hand, the SCR failed to show imagery generalization once acquiring differential fear conditioning to the visual percepts. Broadly, these findings replicate previous research demonstrating that visual fear conditioning can generalize to instances of imagining the corresponding conditioned stimuli. Such imagery generalization has previously been associated with significant differential self-reported fear, SCR and right aIn activity (Greening *et al.*, 2022). It is also consistent with previous research demonstrating the imagery of the conditioned stimuli following visual fear conditioning can be used for extinction learning (Reddan *et al.*, 2018; Jiang and Greening, 2021; Hoppe *et al.*, 2022) and reconsolidation (Agren *et al.*, 2017; Grégoire and Greening, 2019).

One potential concern is that participants are not forming a mental image during imagery trials, and participants are instead conditioning to the auditory cue or to a semantic representation being elicited by the auditory cue. Regarding the first possibility of not forming a mental image, in the self-report questionnaire, participants are asked about the two Gabor patches without reference to CS+ or CS−. Importantly, participants reported significant fear when imagining the CS+ Gabor compared to imagining the CS− Gabor, which they would not have done if conditioning was to some other sensory feature such as the auditory cue. Participants also self-reported generally robust mental imagery vividness for both the CSs, which is consistent with the inference that the participants were generating an imagery percept (Pearson and Kosslyn, 2015). Consistent with this inference, the conjunction analysis involving the imagery trials from the two habituation/practice phases revealed significant activation in brain regions commonly associated with visual imagery (Dijkstra *et al.*, 2017; Moen *et al.*, 2020). Of note, the results included significant activation in the visual cortex, including the occipital lobe and lateral occipital cortex. Moreover, significant activity was observed in this same visual cortex cluster during the fear acquisition runs of both the imagery and visual acquisition phases. Additionally, even if mental imagery involved a semantic representation, the activation of semantic information of concrete objects involves, and may not be dissociable from, the concurrent activation of perceptual information (Wang *et al.*, 2010; Gullick *et al.*, 2013).

Another related potential concern might be that the auditory cues prior to every trial account for the generalization effects; however, this concern was addressed recently using a multi-experiment behavioral study (Burleigh *et al.*, 2022). Specifically, the researchers found inferentially similar generalization results when the auditory cues were present (i.e. experiment 1) *vs* removed from the view trials (auditory cues were always present on imagery trials, i.e. experiments 2 and 3). Notably, the trial structure of the present study is identical to the structure used in experiment 1 of Burleigh *et al.*, (2022), which involved the auditory cue on every trial. Moreover, conditioning to the auditory cues would require differential trace conditioning in the presence of a distracter (i.e. the visual or imagery percept of the Gabor patches). Previous research has demonstrated that differential trace conditioning is highly susceptible to disruption by distraction, in which case we would have predicted to find no significant differential fear acquisition at all (Carter *et al.*, 2003). Although the possibility that participants fear-conditioned exclusively to the auditory cues cannot be ruled out entirely, the present findings along with other recent research findings indicate that the most likely interpretation is that participants generated depictive mental images of the Gabor patches in visual cortices, which were subjectively vivid, and to which participants acquired fear of receiving the shock. Future research could further assess this interpretation by excluding the auditory cue from visual trials, as was done in experiments 2 and 3 of Burleigh *et al*., (2022).

The present findings begin to quantify, both behaviorally and neurally, how mental imagery interacts with emotion-related processes such as the acquisition of differential fear conditioning and the generalization of differential fear conditioning. They are also potentially important for furthering our more general understanding of how mental imagery contributes to both the formation and treatment of psychiatric disorders such as anxiety, PTSD and specific phobias (Lang, 1977; Hackmann and Holmes, 2004; Holmes *et al.*, 2007; Holmes and Mathews, 2010; Pearson *et al.*, 2015; Ji *et al.*, 2016).

## Supplementary Material

nsac063_Supp

## Data Availability

The data underlying this article are available at openeuro.org and can be accessed with the assession number ds004393, or at https://doi.org/10.18112/openneuro.ds004393.v1.0.0.
