# Supplementary material for: Fear in the mind’s eye: the neural correlates of differential fear acquisition to imagined conditioned stimuli
Source: Soc Cogn Affect Neurosci. 2023 Jan 11;18(1):nsac063. doi: 10.1093/scan/nsac063 (PMC10036874; doi:10.1093/scan/nsac063)
Supplement: nsac063_Supp [file nsac063_supp.zip › scan-22-022-File013.docx]

## Supplemental

### 1. Methods

#### 1.1 Instructions given prior to the Imagery Acquisition Phase


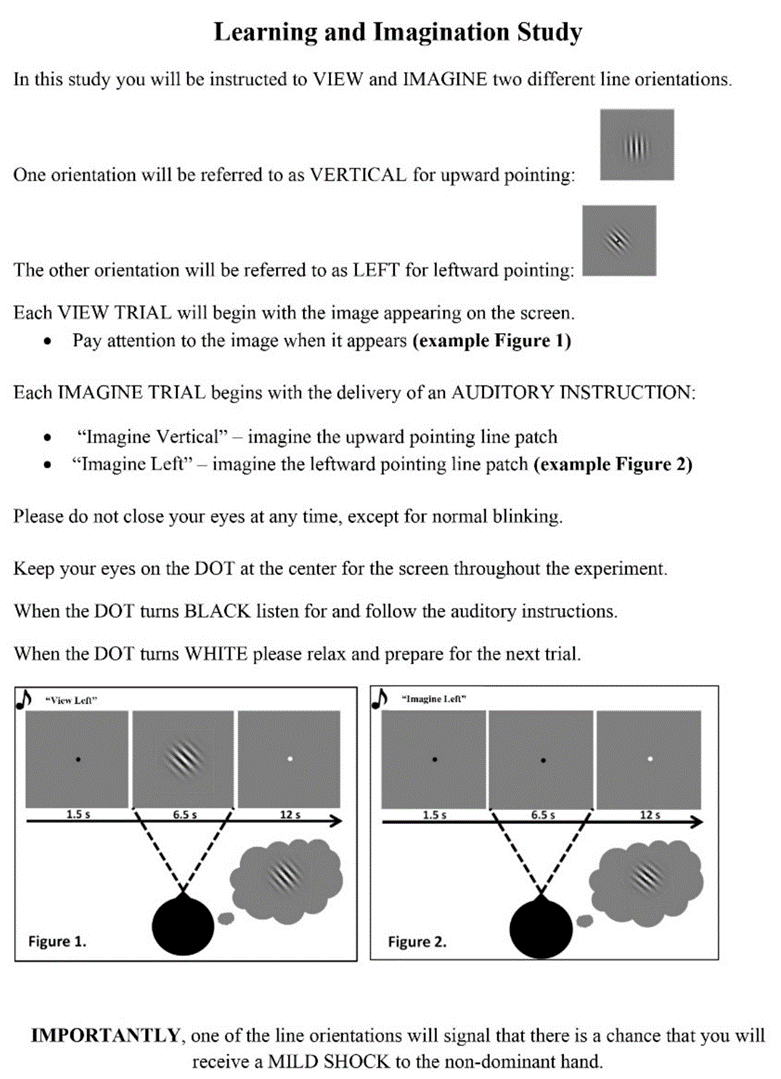


Fig. S1. Each participant was guided through these instructions prior to receiving the Imagery Acquisition phase

#### 1.2 Instructions given prior to Visual Acquisition Phase


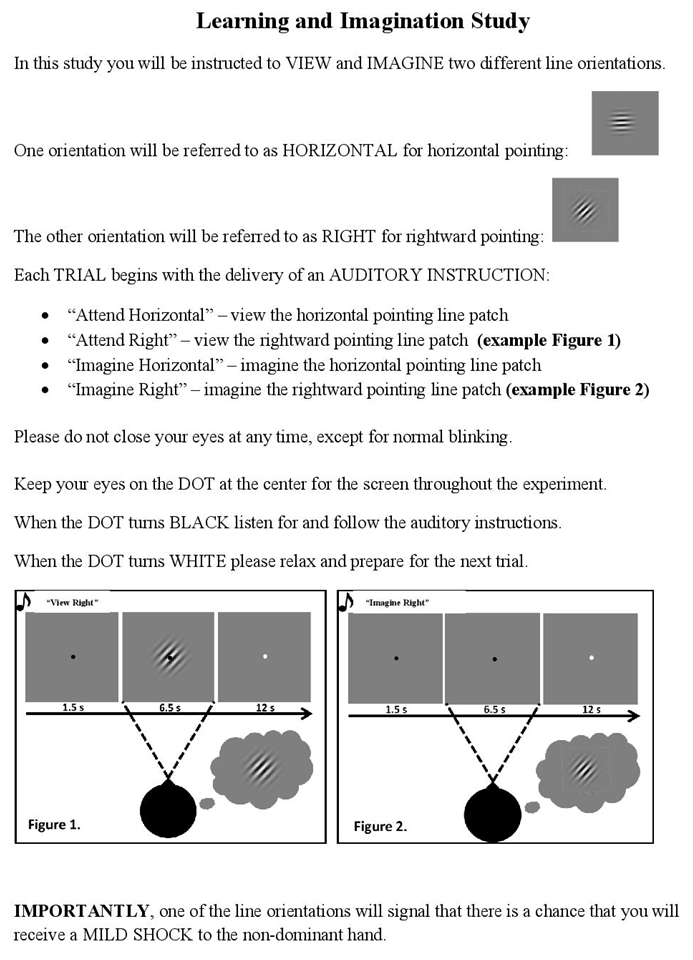


Fig. S2. Each participant was guided through these instructions prior to receiving the Visual Acquisition phase

#### 1.3 Likert-Style Questionnaire Imagery Acquisition


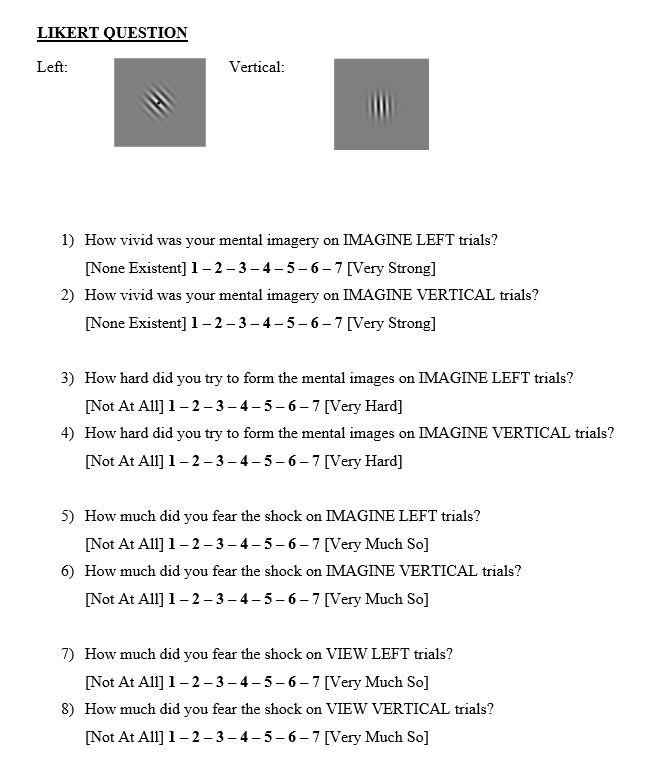


Fig. S3. Each participant answered this self-reported questionnaire after receiving the Imagery Acquisition phase.

#### 1.4 Likert-Style Questionnaire Visual Acquisition

####
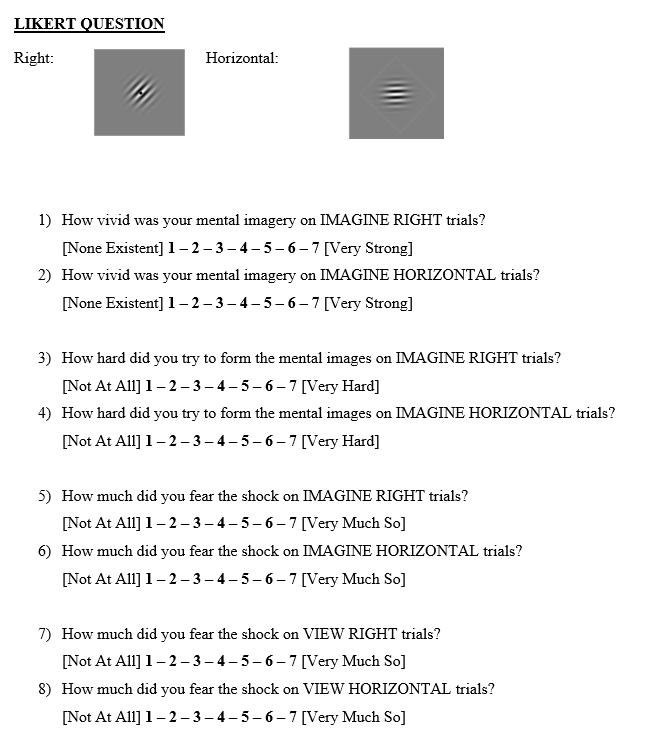


Fig. S4. Each participant answered this self-reported questionnaire after receiving the Visual Acquisition phase.

### 2. Results

#### 2.1 Subjective Questionnaires

|  | Mean | SD |
| --- | --- | --- |
| VVIQ | 64.96 | 7.53 |
| State Anxiety Inventory | 36.87 | 4.76 |
| Trait Anxiety Inventory | 36.13 | 10.81 |
| Attentional Control Scale | 53.60 | 8.59 |

Table S1. Descriptive statistics of questionnaires completed by participants.

#### 2.2 Self-Reported Fear

##### 2.2.1 Likert-style Descriptive Data

|  | Imagery Acquisition Phase | | Visul Acquisition Phase | |
| --- | --- | --- | --- | --- |
|  | Mean | SD | Mean | SD |
| Vividness: CS+ imagine | 5.15 | 1.43 | 4.96 | 1.45 |
| Vividness: CS- imagine | 5.07 | 1.49 | 5.11 | 1.42 |
| Effort: CS+ imagine | 5.11 | 1.58 | 4.89 | 1.48 |
| Effort: CS- imagine | 4.67 | 1.64 | 5.19 | 1.44 |
| Fear: CS+ imagine | 4.52 | 1.78 | 3.15 | 1.92 |
| Fear: CS- imagine | 1.81 | 1.42 | 1.85 | 1.38 |
| Fear: CS+ view | 2.81 | 2.13 | 3.93 | 1.94 |
| Fear: CS- view | 1.37 | 0.97 | 1.89 | 1.37 |

Table S2. Descriptive statistics of self-reported, likert-style questionnaire completed for each phase by participants

#####

##### 2.2.2 Self-Reported Fear ANOVA Results

**
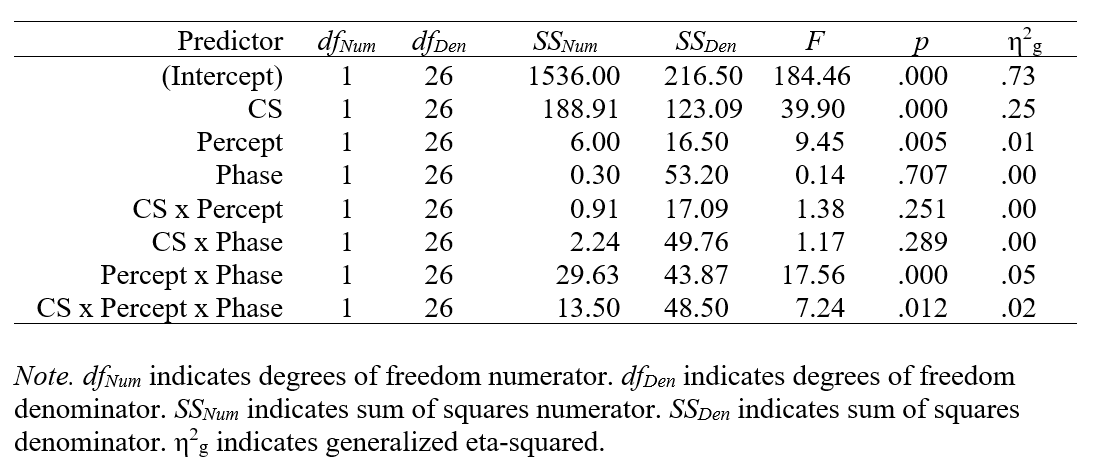
**

Table S3. Full table of ANOVA results using the self-reported data. The 2x2x2 ANOVA consisted of CS (CS+ vs CS-), Percept (viewed stimuli vs imagined stimuli), and Phase (Visual Acquisition Phase vs Visual Acquisition Phase)

##### 2.2.3 Self-Reported Fear ANOVA Results including Order Effect – Imagery Acquisition Phase


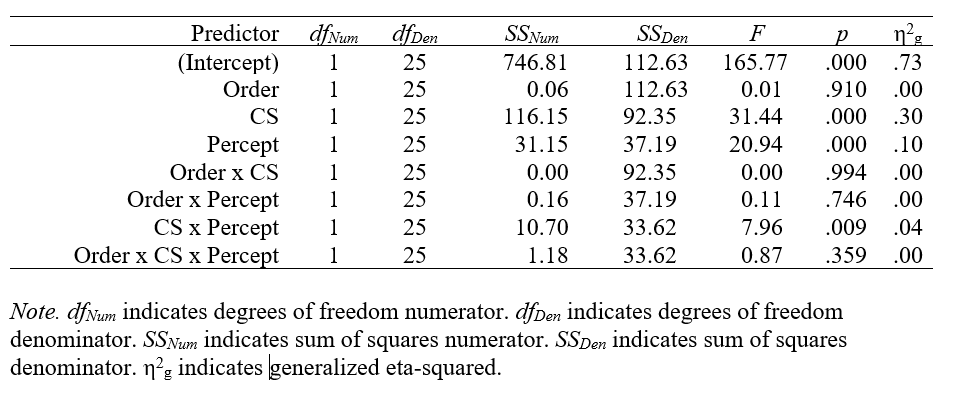


Table S4. Full table of ANOVA results using the self-reported data from the Imagery Acquisition Phase. The Mixed ANOVA consisted of the within factors of CS (CS+ vs CS-) and Percept (viewed stimuli vs imagined stimuli), and the between factor of Order (Visual Acquisition Phase presented first vs Visual Acquisition Phase presented first)

##### 2.2.4 Self-Reported Fear ANOVA Results including Order Effect – Visual Acquisition Phase


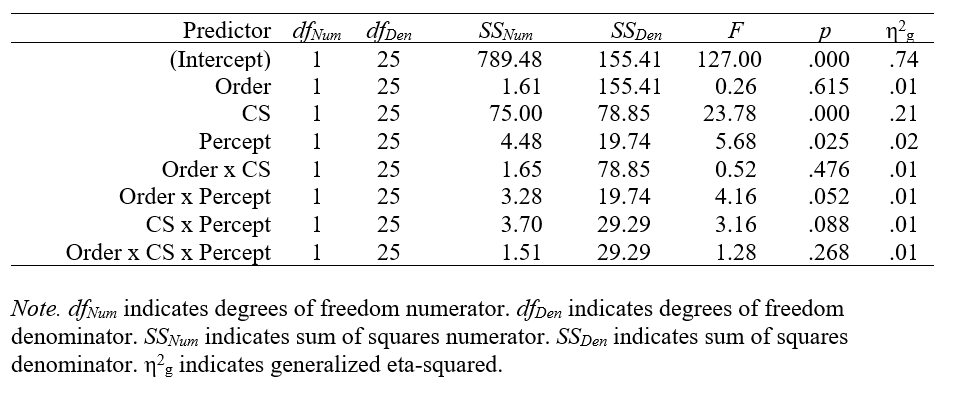


Table S5. Full table of ANOVA results using the self-reported data from the Visual Acquisition Phase. The Mixed ANOVA consisted of the within factors of CS (CS+ vs CS-) and Percept (viewed stimuli vs imagined stimuli), and the between factor of Order (Visual Acquisition Phase presented first vs Visual Acquisition Phase presented first)

##### 2.2.4 Self-Reported Fear ANOVA Results with All Participants

**
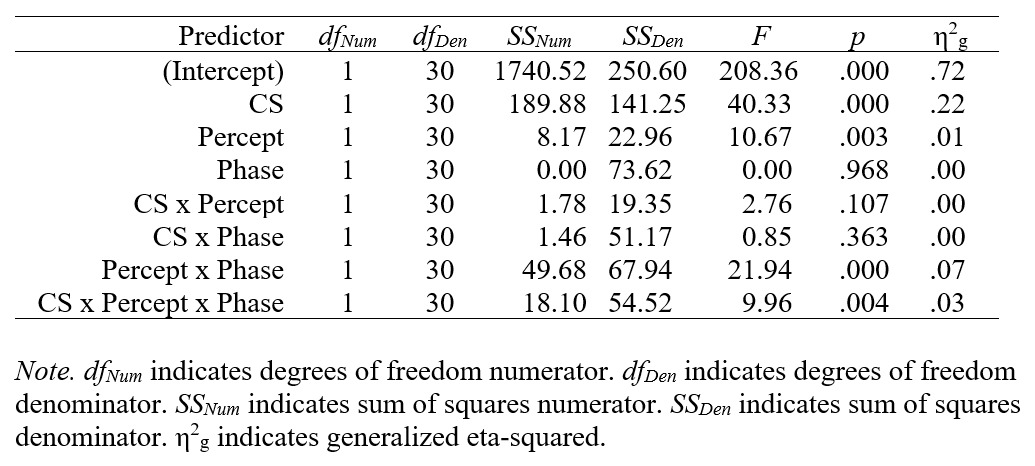
**

Table S5. Full table of ANOVA results using the self-reported data from all participants. This table includes participants whose SCRs were deemed as too noisy or non-responders. The 2x2x2 ANOVA consisted of CS (CS+ vs CS-), Imagery (viewed stimuli vs imagined stimuli), and Set (Visual Acquisition Phase vs Visual Acquisition Phase)

##### 2.2.5 Self-Reported Fear Pairwise Comparison Results

To unpack the three-way interaction, we conducted pairwise comparisons between the various conditions. Here we report those results that are not included in the main manuscript, as they are all non-significant.

There was no significant difference between conditioning to the viewed CS+ (Visual Acquisition phase) and conditioning to the imagined CS+ (Imagery Acquisition phase), *t*(26) = 1.44, *p* = 0.16. There was also no significant difference between the generalized percepts, irrespective of the acquisition phase. The imagined CS+ from the Visual Acquisition phase was not different from the visual CS+ from the Imagery Acquisition phase, *t*(26) = 0.93, *p* = 0.36.

Lastly, we evaluated the CS- conditions’ potential contributions to the three-way interaction and found no significant pairwise difference between the various CS- conditions. In the Visual Acquisition phase there was no significant difference between the imagined CS- and the viewed CS-, *t*(26) = 0.44, *p* = 0.66. In the Imagery Acquisition phase there was also no difference between the imagined CS- and viewed CS-, *t*(26) = , *p* =. Moreover, there was no significant difference between conditioning to a visual CS- (Visual Acquisition phase) and conditioning to an imagined CS- (Imagery Acquisition phase), *t*(26) = 0.34, *p* = 0.74. There was also no significant difference between the generalized percepts, irrespective of the acquisition phase.

In assessing the CS+s, there was no significant difference between imagining and viewing the CS+s in the Visual Acquisition phase, *t*(26) = 2.03, *p* = 0.52.

##### 2.2.6 Bayesian Analysis Self-Reported Fear data

In the Imagery Acquisition phase, the Bayesian Sensitivity Analysis assessing the participants’ self-reported fear when imagining the CS+ and imagining the CS- indicates that this conclusion is likely very reliable, BF_10_(0.707) = 9968.59, BF_10_(1) = 10827.27, and BF_10_(1.41) = 10587.50. Evaluation of the generalization of differential conditioning when viewing the CS+ compared to viewing the CS- resulted a sensitivity analysis of BF_10_(0.707) = 23.59, BF_10_(1) = 21.38, and BF_10_(1.41) = 17.86., indicating modest reliability.

In the Visual Acquisition phase, the Bayesian Sensitivity Analysis assessing the participants’ fear when viewing the CS+ versus viewing the CS- resulted in: BF_10_(0.707) = 429.75, BF_10_(1) = 429.83, and BF_10_(1.41) = 389.01. The Sensitivity Analysis assessing the participants’ fear when imagining the CS+ compared to imagining the CS- resulted in: BF_10_(0.707) = 22.02, BF_10_(1) = 19.91, and BF_10_(1.41) = 16.5.

#### 2.3 Vividness Ratings

**
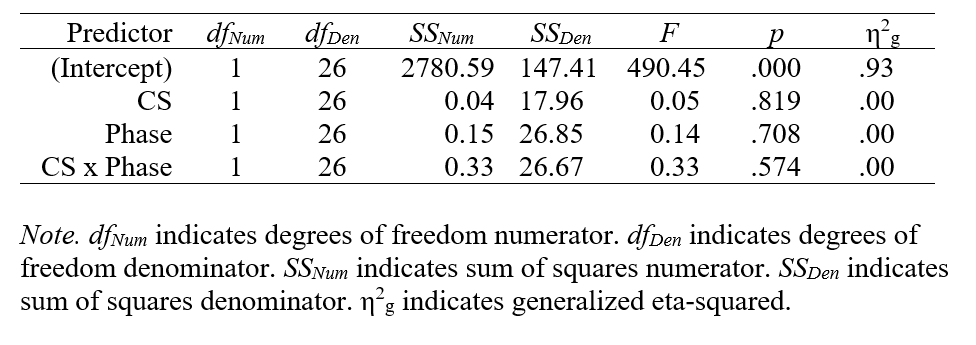
**

Table S5. Full table of ANOVA results using the vividness data. The 2x2 ANOVA consisted of CS (CS+ vs CS-) and Phase (Visual Acquisition Phase vs Visual Acquisition Phase)

#### 2.4 Effort Ratings

**
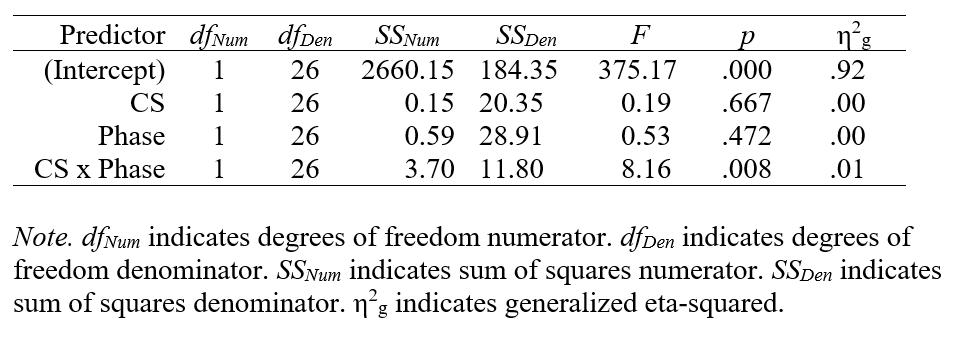
**

Table S6. Full table of ANOVA results using the effort data. The 2x2 ANOVA consisted of CS (CS+ vs CS-) and Phase (Visual Acquisition Phase vs Visual Acquisition Phase)

#### 2.5 SCR Data

##### 2.5.1 SCR Descriptive Data

|  | Imagery Acquisition | | Visual Acquisition | |
| --- | --- | --- | --- | --- |
|  | Mean | SD | Mean | SD |
| CS+ Imagine | 0.38 | 0.39 | 0.20 | 0.21 |
| CS- Imagine | 0.22 | 0.22 | 0.18 | 0.16 |
| CS+ View | 0.25 | 0.23 | 0.36 | 0.35 |
| CS- View | 0.21 | 0.22 | 0.20 | 0.17 |

Table S7. Descriptive statistics of SCR data of participants during both the Visual Acquisition phase and Imagery Acquisition phase

##### 2.5.2 SCR ANOVA Results

**
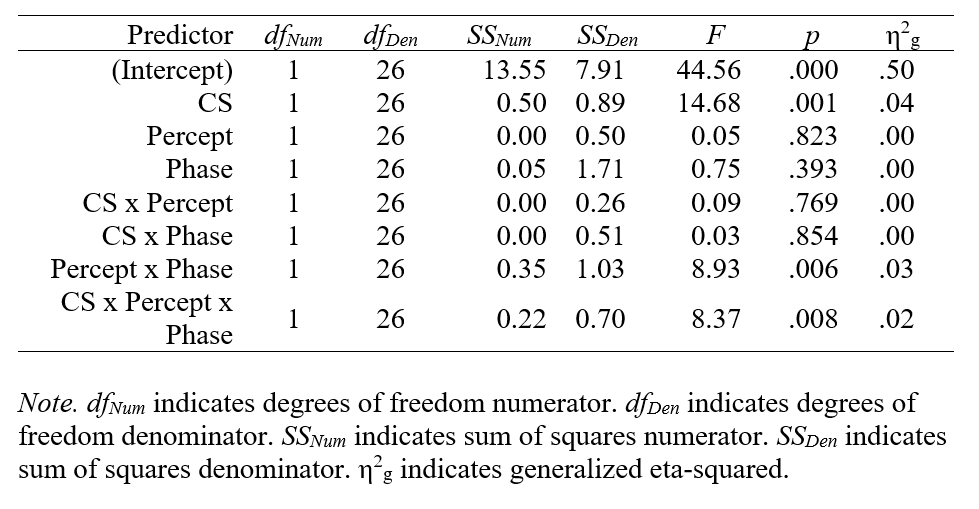
**

Table S8. Full table of ANOVA results using the SCR data. The 2x2x2 ANOVA consisted of CS (CS+ vs CS-), Percept (viewed stimuli vs imagined stimuli), and Phase (Visual Acquisition Phase vs Visual Acquisition Phase)

##### 2.5.3 SCR ANOVA Results including Order Effect – Imagery Acquisition Phase


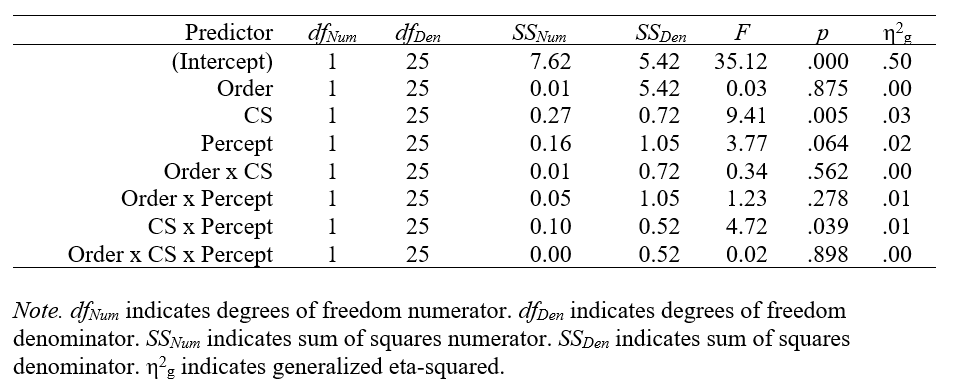


Table S9. Full table of ANOVA results using the SCR data from the Imagery Acquisition Phase. The Mixed ANOVA consisted of the within factors of CS (CS+ vs CS-) and Percept (viewed stimuli vs imagined stimuli), and the between factor of Order (Visual Acquisition Phase presented first vs Visual Acquisition Phase presented first)

##### 2.5.4 SCR ANOVA Results including Order Effect – Visual Acquisition Phase


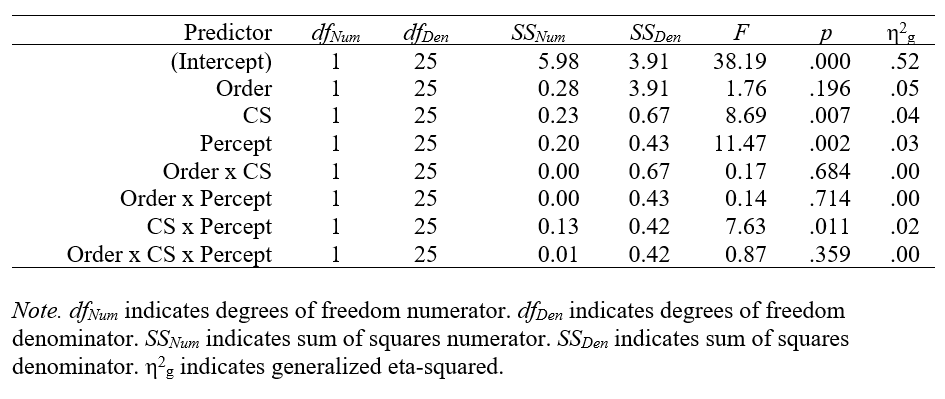


Table S10. Full table of ANOVA results using the SCR data from the Visual Acquisition Phase. The Mixed ANOVA consisted of the within factors of CS (CS+ vs CS-) and Percept (viewed stimuli vs imagined stimuli), and the between factor of Order (Visual Acquisition Phase presented first vs Visual Acquisition Phase presented first)

##### 2.5.5 SCR Pairwise Comparison Results

In order to unpack the three-way interaction we conducted pairwise comparisons between the various conditions. Here we report those results that are not included in the main manuscript, as they are all non-significant.

Conditioning to the viewed CS+ in the Visual Acquisition phase was not significantly different from conditioning to the imagined CS+ in the Imagery Acquisition phase, *t*(26) = 0.42, *p* = 0.68. There was also no significant difference in SCR between the generalized percepts irrespective of the acquisition phase. The SCR to the imagined CS+ from the Visual Acquisition phase was not significantly different from that of the viewed CS+ from the Imagery Acquisition phase, *t*(26) = 1.00, *p* = 0.32.

Within the Visual Acquisition phase, the viewed CS- was not significantly different from the imagined CS-, *t*(26) = 1.00, *p* = 0.33. In the Imagery Acquisition phase the imagined CS- was not significantly different from the imagined CS-, *t*(26) = 0.44, *p* = 0.66.

The CS- conditions produced no significant difference between the imagined CS- from the Visual Acquisition phase and the viewed CS- from the Imagery Acquisition phase, *t*(26) = 0.66, *p* = 0.51. There was also no significant difference in the SCR between conditioning to a CS- view from Visual Acquisition phase versus generalizing to a CS- view from Imagery Acquisition phase, *t*(26) = 0.26, *p* = 0.80. Similarly, the conditioned response to a CS- imagine in the Imagery Acquisition phase was not significantly different from the generalization response to a CS- imagine from Visual Acquisition phase, *t*(26) = 1.36, *p* = 0.18. Likewise, there was no significant difference in SCR between the generalized percepts irrespective of the acquisition phase. The SCR to the CS- imagine from the Visual Acquisition phase was not significantly different from the SCR to the CS- view from Imagery Acquisition phase, *t*(26) = , *p* =. The SCR to the CS- view from the Visual Acquisition phase was also not significantly different from the SCR to the CS- imagine from Imagery Acquisition phase, *t*(26) = 0.71, *p* = 0.49.

Viewing the CS+ in the Imagery Acquisition phase was also not significantly different from viewing the CS+ in the Visual Acquisition phase, *t*(26) = 1.84, *p* = 0.08.

##### 2.5.6 Bayesian Analysis SCR data

In the Imagery Acquisition phase, the Bayesian Sensitivity Analysis assessing the participants’ SCR when imagining the CS+ versus imagining the CS- resulted in: BF_10_(0.707) = 12.56, BF_10_(1) = 11.11, and BF_10_(1.41) = 9.11. The Sensitivity Analysis assessing the participants’ fear when viewing the CS+ and viewing the CS- resulted in: BF_01_(0.707) = 0.38, BF_01_(1) = 0.28, and BF_01_(1.41) = 0.21.

In the Visual Acquisition phase, the Bayesian Sensitivity Analysis of the SCR data for viewing the CS+ versus viewing the CS- resulted in: BF_10_(0.707) = 8.54, BF_10_(1) = 7.44, and BF_10_(1.41) = 6.03. The Sensitivity Analysis for imagining the CS+ versus imagining the CS- resulted in: BF_10_(0.707) = 0.40, BF_10_(1) = 0.30, and BF_10_(1.41) = 0.22.

#####
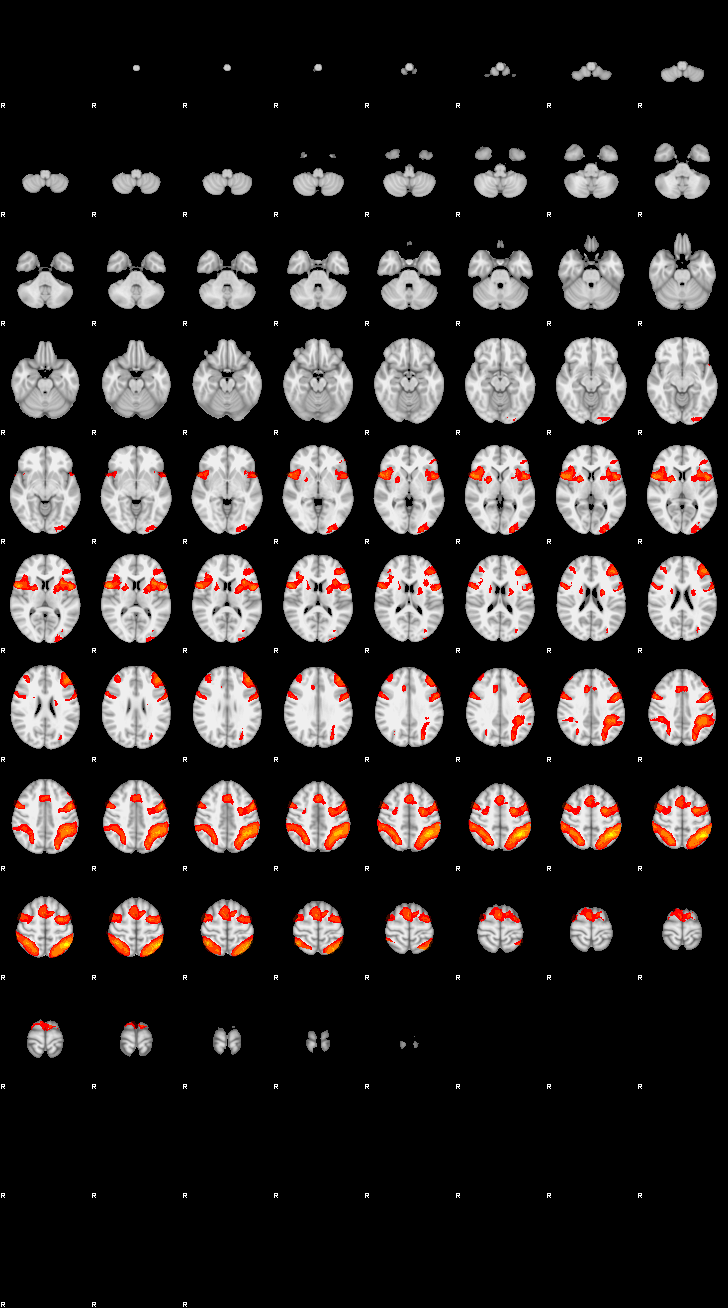
2.6.1 Whole-brain, corrected conjunction fMRI results

Whole-brain results from the conjunction analysis involving imagery trials versus baseline from the habituation/practice runs of both the imagery acquisition phase and the visual acquisition phase.
